# Validity and reliability of the Greek version of Implementation Leadership Scale (ILS)

**DOI:** 10.1186/s40359-020-00413-5

**Published:** 2020-05-14

**Authors:** Eleni Mandrou, Andreas Tsounis, Pavlos Sarafis

**Affiliations:** 1 Hellenic Open University, Patra, Greece; 2 Aristotle University of Thessaloniki, School of Psychology, Thessaloniki, Greece; 3 School of Health Sciences, Department of Nursing, Vragadinou Str, 3041 Limassol, Cyprus

**Keywords:** Implementation, Leadership, Validity, Reliability, Greece

## Abstract

**Background:**

The need for developing pragmatic and reliable measures that affect evidence-based practice has been highlighted in organizational studies. The aim of the current study is to evaluate the psychometric properties of the Greek version of Implementation Leadership Scale (ILS). ILS is a brief and effective tool for measuring leadership when implementing evidence based practices.

**Methods:**

The translation process followed World Health Organization guidelines. Face and content validity were examined. Then, the psychometric properties of ILS were tested with a sample of 143 nurses and midwifes working in a private Greek hospital. Confirmatory Factor Analyses for structural validity testing, Pearson coefficient for convergent and discriminant validity testing as well as internal consistency analysis for reliability testing were conducted. Quality of leadership scale from COPSOQ II and Organizational Climate Measure were used for assessing convergent and discriminant validity, respectively.

**Results:**

Greek version of ILS show good face and content validity. CFA results (x^2^ = 100. 69 (50); CFI = 0.93; GFI = 0.83; RMSEA = 0.06) confirmed the four-factor structure of the scale (Proactive, Knowledgeable, Supportive and Perseverant leadership). The internal consistency was excellent (a = 0.94 for total scale and between 0.85 and 0.91 for subscales). Analyses also revealed good convergent and discriminant validity.

**Conclusions:**

The findings suggest that the Greek Version of ILS is a valid and reliable tool for measuring leadership of evidence based practices implementation. However, further research for assessing its psychometric properties in various samples and more professional groups is suggested.

## Background

Evidence Based Practices (EBP) across healthcare settings refers to the integration of the best research findings, professionals’ expertise and patient’s unique circumstances and values [1]. EBP implementation in healthcare started in the 1990s, today it is widely accepted due to the great amount of scientific information, new technologies, ageing of the population and rising patient expectations [1]. Moreover, literature suggests that EBP implementation may lead to better healthcare, the patient experience improvement and the reduction of costs, while at the same time improves work-life aspects for the clinicians [2–4].

However, although EBP implementation across healthcare settings has been recognized as an issue of great importance for the quality of healthcare provided and the organizational context improvement, there are still gaps in the development of measures of EBP implementation [5]. The assessment of EBP applications is not only related to clinical outcomes and targeted interventions. One issue of great importance is the development of tools regarding organizational factors and constructs like leadership [6].

Leadership is amongst the most important components of the organizational process and a key factor for the strengthening performance of healthcare systems and units. Leadership styles, especially transformational leadership, may influence employee’s motivation, teamwork and job satisfaction [7], while at the same time may affect issues such as EBP implementation [8]. Evidence suggest that transformational leadership leads to the development of innovation, promotion of EBP, rising from the use and practice of EBP guidelines, positive team functioning and psychological safety of the personnel [6].

Due to the need for the development of pragmatic and reliable measures that affect EBP in organizational studies, Aarons, Ehrhart and Farahnak developed the Implementation Leadership Scale (ILS) [5]. ILS evaluates the behaviors that leaders can perform to support targeted EPB efforts. More specifically, it assesses four aspects of leadership: (i) Proactive leadership that is referred to the degree to which the supervisor establishes clear plans and removes obstacles concerning EBP implementation, (ii) Knowledgeable leadership that describes the degree to which a supervisor is informed about EBP implementation and is able to address specific personnel’s questions, (iii) Supportive leadership that assesses the level of supportiveness and recognition of the staff efforts concerning EBP and (iv) Perseverant leadership that refers to the supervisor’s efforts to persevere in EBP through the ups and downs of the implementation procedure [5]. ILS is a (unit level tool) that focuses in the first-level leadership. So, through the use of this tool the employees assess their immediate group supervisors that manage the day-to-day EBP implementation issues and not the upper-level leaders that mainly set the strategic decisions for an organization [5].

The aim of the current study was to translate and evaluate the psychometric properties of the Greek Version of ILS, which is a brief and effective tool for measuring leadership as far EBP implementation.

## Methods

### Participants and procedure

A total of 160 questionnaires were distributed to nurses and midwifes working in a private general Obstetrics and Gynecology hospital in Greece. The final sample consisted of 143 participants (Response rate: 89.38%). The participants completed the questionnaires at the time of recruitment. They were informed about the aim of the study and that they could terminate their participation at any time without any consequences and that the data would be treated confidentially. Returning the questionnaire was interpreted as informed consent. The data were collected in a period of 3 months in 2018.

### Ethics approval

Our study protocol was submitted and approved by the Ethics Committee of the School of Social Sciences of the Hellenic Open University (Registration number: 10–11/2017). The study was also submitted and approved by the Scientific Council of the Private Hospital “Hygeia A.E” in Athens (Registration number: 10–2/2018). All participants provided written informed consent.

### Instruments

#### Implementation leadership scale (ILS)

The ILS is comprised of four factors including: (i) Knowledgeable leadership, (ii) Proactive leadership, (iii) Supportive leadership and (iv) Perseverant leadership [5]. Each factor is assessed with three items ranked on a 5-point scale from 0 (not at all) to 4 (to a very great extent) indicating the degree to which the supervisor performs the above behaviors. Total ILS score derives from the mean of the subscales. Total ILS score ranges from 0 to 48, while score for each sub-scale ranges from 0 to 12. Regarding the interpretation of the scale results, there are no cut-off scores. ILS items are described in Table 2.

#### Quality of leadership scale

The Quality of leadership dimension of the Copenhagen Psychosocial Questionnaire Version II (COPSOQ II) was used for the convergent validity assessment [9]. The initial version of the COPSOQ was developed as a tool covering a broad range of psychosocial factors, including most of the main studies dimension of occupational health psychology like job insecurity, job demands, role clarity, social support from colleagues and supervisors and possibilities for development [9]. COPSOQ II is an expanded version of the initial which was developed in order to incorporate aspects arising from the experience of use of the initial COPSOQ [9]. In total is comprised from 24 dimensions (92 items). The Quality of leadership dimension which was used in our study is comprised from seven items such as: “To what extent would you say that your immediate superior appreciates the staff and shows consideration for the individual” and “... is good at allocating the work”. Items ranked in a 5-point scale from 1 (to a very small extent) to 5 (to a very large extent). Scale score ranges from 7 to 35, while there are no cut off scores indicating high or low leadership quality. Cronbach’ s alpha of the scale was .95.

#### Organizational climate measure

The Organizational Climate Measure was developed by Patterson et al. [10] and it is based upon Quinn and Rohrbaugh’s Competing Values Model [11] was used for discriminant validity assessment. It is consisted from 17 subscales that describe the main four domains of competing values framework: (i) human relations, (ii) internal process, (iii) open systems and (iv) rational goal. In our study we used four subscales: (i) autonomy (from human relations domain) (5 items), (ii) formalization (from internal process) (5 items), (iii) efficiency (4 items) and (iv) performance feedback (from rational goal domain) (5 items). Items ranked in a 4-point scale from 1 (total false) to 4 (total true). Regarding autonomy, formalization and performance feedback score ranges from 5 to 20, while for efficiency score ranges from 4 to 16.Similarly to the previous scales there are no cut-off scores. Cronbach’ s alpha of the sub-scales were: 0.68 for autonomy, 0.71 for formalization, 0.62 for efficiency and 0.72 for performance feedback.

### Translation procedure

Translation of the original ILS into Greek was carried out by a translation/back-translation procedure which is the most common applied process for inventories [12]. Our approach was based on the systematic approach of World Health Organization regarding translation and adaptation of research instruments [13]. Namely, forward translation, panel meeting, backward translation, pre-testing and final consensus were included in the process. In the first step two professional translators performed the forward translation of the ILS into Greek. In the second step, the forward translation drafts were checked by the three authors and one independent researcher, who checked and discuss the discrepancies between both two translations. At the end of this panel meeting a single translation of the ILS was agreed upon. In the third step, a backward translation was made by two other translators. The backward translations were compared with the initial English version for the identification of discrepancies. Since no discrepancies were identified between the back-translation and the original ILS English version a final agreement upon Greek ILS was reached. In the fourth step (pre-testing) the final Greek version was given to 9 volunteer participants for pilot testing and checking for its’ clarity and understandability. Participants filled out the scale and then they answered a number of questions about the general comprehensiveness of the instrument and the clarity and ambiguity of each separate item (e.g., if there was a word or an expression that they could not understand, what they thought that each question was asking, if they could repeat questions in their own words). In the fifth step, final corrections and alterations were made. Namely, based on the comments of the translators and the participants of the pilot study, we decided to make some slight corrections in order to improve the clarity of wording and to add a statement in the initial part of the tool (before items) clarifying the meaning of EBP, since in Greece the use of the specific terminology in not as common as in other countries.

Face validity of the scale was tested by the three researchers (authors) after discussing the comments of the translators and the participants of the pilot study. All comments discussed until a consensus was reached regarding the final scale version after all the corrections that were made in the fifth step.

**Fig. 1:**
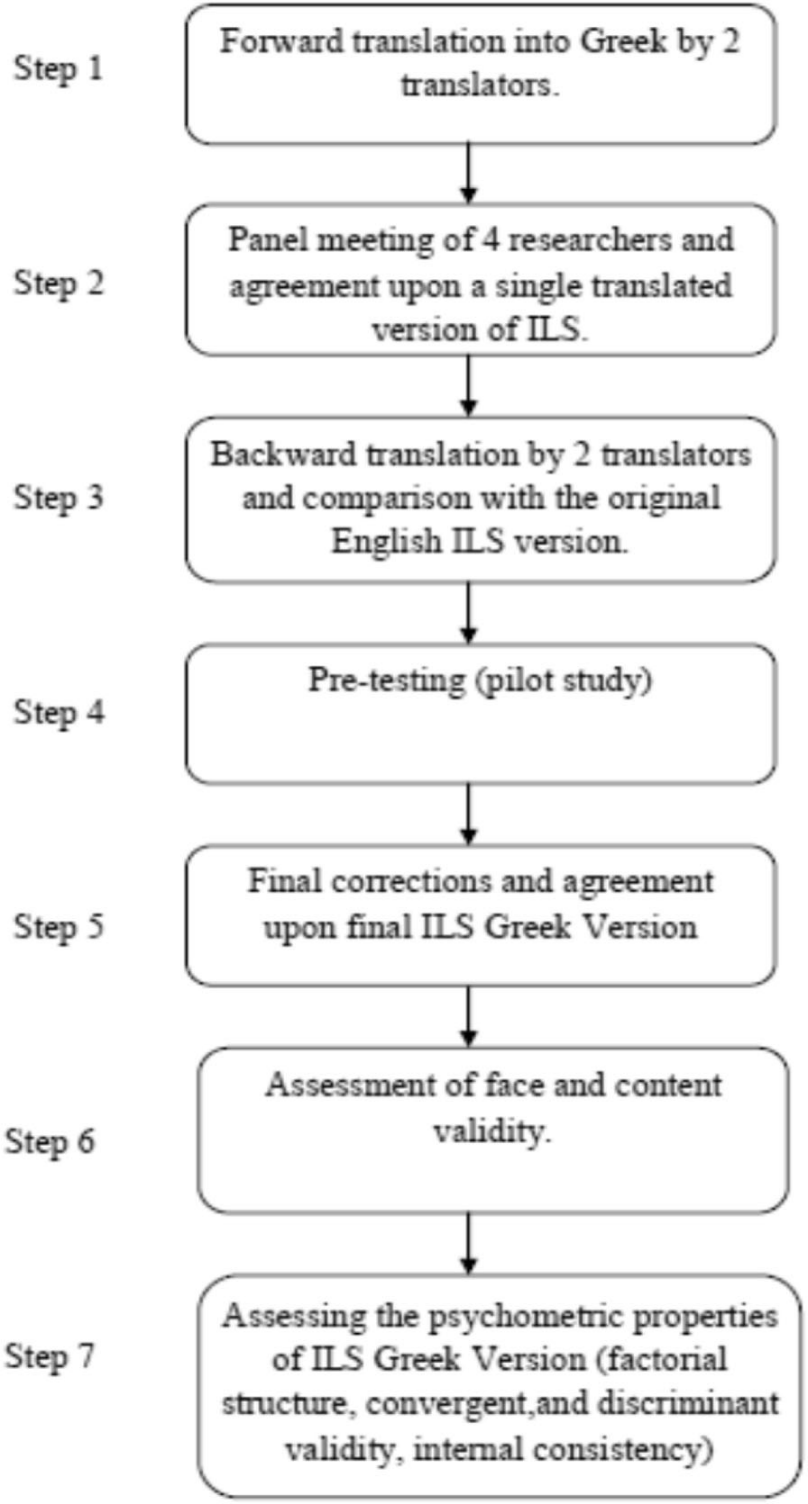
Translation, adaptation and psychometric validation process

Content validity was examined from the three authors and four independent researchers with relevant research experience, using the content validity index (CVI) [14]. Namely, each one of the seven researchers rated each ILS item on its relevance using a 4-point scale ranging from (4) “highly relevant” to (1) “not relevant”. Then, by dividing the number of those panelists that rated 3 or 4 to the total number of panelist, CVI was calculated. CVI > .80 indicates appropriateness [14]. The steps that were followed for the translation, adaptation and psychometric validation of ILS are presented in Fig. 1.

The same procedure (forward/backward translation) until the third step was also followed for quality of leadership dimension of COPSOQ II [9] and the four sub-scales of Organizational Climate Measure [10], that are not validated in Greece and they were used for the examination of convergent and discriminant validity.

### Statistical analysis

Confirmatory Factor Analysis (CFA) was applied to test how well the dimensions of ILS fit the data. The fit of the model was assessed with the comparative fit index (CFI), the goodness of fit index (GFI) and the root mean square error of approximation (RMSEA) [15]. For CFI and GFI, values close or greater to 0.95 show good data fit and for RMSEA values less than 0.05 indicate good fit, while values as high as 0.08 indicate a reasonable fit [16]. Pearson coefficients were used to explore convergent and discriminant validity. Cronbach alpha and Guttman Split Half coefficients were applied to estimate internal consistency. Data were analyzed using SPSS software (Statistical Package for the Social Sciences, version 20; IBM, Armonk, NY, USA) and AMOS (SPSS, Chicago, IL, USA) statistical software.

## Results

### Face and content validity

After the minor suggestions, concerning clarity of wording, that were given from the participants of the pilot study and the researchers of the panel meeting and the addition of the statement clarifying the meaning of EBP, no major remarks were emerged. So, the authors agreed that Greek version of ILS had good face validity. As far as the content validity, all 12 items of ILS achieved a CVI rating between 0.80 and 1.00. So, the content validity was also satisfactory.

### Sample characteristics and descriptive statistics

The final sample consisted of 143 nurses and midwifes. Most of the participants were females (91.6%, *N* = 131), with mean age 35.4 years (SD = 7.7) and 11.8 years (SD = 7.8) as a mean score of professional experience. Most of them 58.9% (*N* = 83) were midwifes, while, regarding family status, the majority of them (43.7%, *N* = 62) was married (Table 1).

**Table 1:** Socio-demographic features of the sample

	N	%	Mean	***SD***
**Gender**				
Men	12	8.4		
Women	131	91.6		
Age			35.4	7.7
**Family status**				
Unmarried	62	43.7		
Married	72	50.7		
Divorced	6	4.2		
Widowed	2	1.4		
**Educational Level**				
High School	10	7.0		
Technical School	33	23.2		
University	91	64.1		
Post-graduate studies	8	5.6		
**Profession**				
Midwife	83	58.9		
Nurse	58	41.1		
**Working department**				
Surgery	39	27.7		
Delivery room	26	18.4		
Intensive Care Unit	25	17.7		
Clinics	51	36.2		
**Working experience**			11.8	7.8
**Working in current position**			9.6	7.5

Descriptive statistics of the ILS are presented in Table 2. The total scale mean was 3.19 (SD = 0.56), while means for the four subscales ranged from 3.07 to 3.39.

**Table 2:** Means, Standard Deviations Cronbach alpha’ s and alpha’ s if item deleted of the ILS

ILS items and subscales	Mean	SD	a	a (if item deleted)
**1.Proactive leadership**	3.07	0.56	0.85	
Removed obstacles to implementation of EBP	2.99	0.68		0.93
Established clear standards for implementation of EBP	3.09	0.71		0.93
Developed a plan to facilitate EBP implementation	3.15	0.53		0.93
**2. Knowledgeable leadership**	3.39	0.57	0.88	
Is knowledgeable about EBP	3.44	0.60		0.93
Is able to answer staff questions about EBP	3.37	0.62		0.93
Knows what he/she is taking about when it comes to EBP	3.37	0.67		0.93
**3. Supportive leadership**	3.16	0.80	0.91	
Supports employee efforts to learn more about EBP	3.22	0.89		0.93
Recognizes and appreciates employee efforts	3.08	0.90		0.93
Supports employee efforts to use EBP	3.18	0.80		0.93
**4. Perseverant leadership**	3.18	0.66	0.86	
Perseveres through the ups and downs of implementing	3.20	0.74		0.93
Carries on through the challenges of implementing EBP	3.20	0.72		0.93
Reacts to critical issues regarding implementation of EBP	3.14	0.78		0.93
**ILS total**	3.19	0.56	0.94	

### Reliability

Cronbach alpa coefficient for the four dimensions of the ILS ranged from 0.85 to 0.91, while the reliability estimate for the total scale was 0.94 (Table 2). Split-half was also done by dividing the measure in two halves. Guttman Split Half coefficient was 0.85.

### Factorial validity

**Fig. 2:**
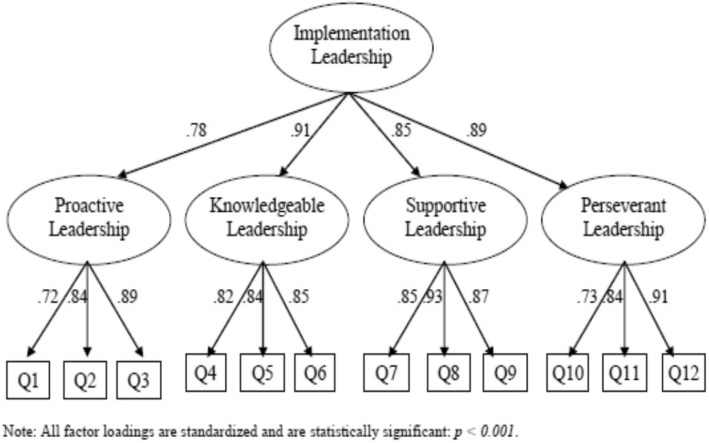
Second-order confirmatory factor analysis and factor loadings of Greek version of ILS

CFA was used to confirm the already designed structure of the ILS. The CFA results indicated that the proposed four facets’ model fit the data well (x^2^ = 100. 69; df = 50; *p* < .001. More specifically, the comparative fit index (CFI), the goodness of fit index (GFI) and the root mean square error of approximation (RMSEA) were equal to 0.93, 0.83 and 0.06, respectively. As presented in Fig. 2, all standardized factor loadings were significant, ranging from for first-order factor loadings and for second-order factor loadings.

### Convergent validity

Quality of leadership scale was given to estimate convergent validity. Pearson correlations between ILS and Quality of leadership scale are presented on Table 3. As predicted, ILS has moderate to high positive correlations with Quality of leadership, but not so much high as to suggest that the two tools measure identical constructs. The correlations between the two scales indicate that aspects of leadership that Quality of leadership scale measure, like effectiveness at communicating with the staff, high priority to further training and personnel planning and consideration for the employee are important for the effective EBP implementation.

**Table 3:** Pearson correlations of ILS with Quality of Leadership and Organizational Climate Measure (OCM) scales and sub-scales

	Implementation Leadership Scale (ILS)				
	Proactive	Knowledgeable	Supportive	Perseverant	Total
**Quality of Leadership**	.590**	.619**	.752**	.731**	.792**
**Organizational Climate (OCM)**	.502	.838	.254	.840	.552
Autonomy	.102**	.154	.229**	.147	.091**
Formalization	.325**	.281**	.283**	.309**	.362**
Efficiency	.233**	.267**	.166*	.250**	.253**
Feedback	.307**	.180*	.116	.054	.175*

* *p* < 0.05**p < 0.01

### Discriminant validity

Pearson correlations between ILS and Organization Climate measure, which was given for discriminant validity estimation, are presented on Table 3. ILS total and all subscales scores had low correlations with formalization and efficiency facets of Organization Climate measure, while total ILS and some of its’ subscales had also positive correlations with feedback and autonomy facets of organizational climate.

## Discussion

The purpose of our study was the translation of ILS into Greek and the examination of its psychometric properties. Concerning the factorial structure and validity, the CFA results indicated that the four-factor model of the Greek version is well adapted and consistent with the original version of the tool. RMSEA value was near to 0.05 while values of CFI and GFI were near to 0.08 indicating a reasonable fit [16]. The results of our study, that was conducted amongst nurses and midwives, are in line with the findings of other studies conducted amongst employees on community-based organizations that provide child welfare services [17], employees in substance abuse treatment organizations and employees in mental health sector [18] and education sector [19]. ILS has also been translated, validated and tested for its psychometric properties in Chinese language [20, 21]. The results of the study in China which was conducted among 234 nurses showed also good model fit index with similar to our study factor loadings that ranged from o.79 to 0.95 and adequate reliability (0.86 to 0.95 for each ILS factor) [20].

The results of convergent validity revealed moderate to high positive correlations but not in level as high as to suggest that quality of leadership is the same construct with leadership that enhance the implementation of EBP. Indeed, although leadership targeting in implementation of EBP include aspects like effective communication with the employees, giving high priority to further training that are criteria of high quality leadership [22–24], implementation leadership is more centered to EBP enhancing in a more practical and specific targeted way [6].

Regarding discriminant validity, as predicted ILS total and all subscales scores had low correlations with formalization and efficiency facets of Organization Climate measure which as a finding is similar to the study of developing and validating of ILS [5]. Additional total ILS and some of its facets had low positive correlations with feedback autonomy facets of organizational climate. The above indicate support for the dicriminant validity hypothesis.

Finally, as far as reliability and internal consistency, the Cronbach’ s alpha values ranged from 0.85 to 0.91 for the subscales while for the total scale was 0.94. The findings also show a high value of split-half reliability. According to the literature, values over 0.70 are considered to be accepted [25]. However, these high values may suggest unidimensionality [26].

### Study limitations

The current study has certain limitations. First, the sample size was relatively small. Second, the data collected only from nurses and midwifes and not employees of other specialties. Third, all the participants worked in the same hospital. So, due to the second and third limitation there was no variation of participants. Fourth, the test-retest reliability was not examined.

### Study implications

Our findings contribute to the potentiality of more reliable assessment of EBP in Greece, since ILS is considered as a pragmatic tool for evaluating the behaviors that leaders can perform to support EPB efforts. Moreover, it may add to the literature by testing the psychometric properties of ILS on other languages beyond English and Chinese and comparing the validity and reliability of the tool in different cultural environments. Keeping the limitations of our study in mind, future studies may further investigate the ILS psychometric properties and use ILS for assessing leadership regarding EBS in organizational studies in Greece.

## Conclusions

The findings of the study suggest that the Greek Version of ILS Scale is a valid and reliable tool for measuring leadership of evidence based practices implementation in Greek healthcare settings. However, further research evaluating the properties of ILS amongst different professional groups of clinicians like doctors and social scientists (e.g. psychologists and social workers) is proposed. Additionally, studies regarding the validity and reliability measurement in a greater variety of healthcare services beyond general hospitals, like mental health units, substance abuse treatment programs, units providing care in elderly and disabled individuals in the community, would be helpful to the direction of the generalization potential of the ILS use. Finally, future research may take under consideration the high values regarding separate sub-scales internal consistency, searching for potential redundancies or unidimensionality of the scale.

### Availability of data and materials

Data can be available from the corresponding author upon reasonable request.

## Data Availability

You may contact with the corresponding author for more information

## Abbreviations

AD: Average Deviation
CFA: Confirmatory Factor Analysis
CFI: Comparative Fit Index
COPSOQ: Copenhagen Psychosocial Questionnaire
CVI: Content Validity Index
EBP: Evidence Based Practices
GFI: Goodness of Fit Index
ILS: Implementation Leadership Scale
RMSEA: Root Mean Square Error of Approximation
